# In a Real-Life Setting, Direct-Acting Antivirals to People Who Inject Drugs with Chronic Hepatitis C in Turkey

**DOI:** 10.5152/tjg.2022.21834

**Published:** 2022-11-01

**Authors:** Figen Sarıgül Yıldırım, Ülkü Üser, Nagehan Didem Sarı, Behice Kurtaran, Yusuf Önlen, Ebubekir Şenateş, Alper Gündüz, Esra Zerdali, Hasan Karsen, Ayşe Batırel, Rıdvan Karaali, Rahmet Güner, Tansu Yamazhan, Şükran Köse, Nurettin Erben, Nevin İnce, İftihar Köksal, Nefise Çuvalcı Öztoprak, Gülşen Yörük, Süheyla Kömür, Tayibe Bal, Sibel Kaya, İlkay Bozkurt, Özgür Günal, İlknur Esen Yıldız, Dilara İnan, Şener Barut, Mustafa Namıduru, Selma Tosun, Kamuran Türker, Alper Şener, Kenan Hızel, Nurcan Baykam, Fazilet Duygu, Hürrem Bodur, Güray Can, Hanefi Cem Gül, Ayşe Sağmak Tartar, Güven Çelebi, Mahmut Sünnetçioğlu, Oğuz Karabay, Hayat Kumbasar Karaosmanoğlu, Fatma Sırmatel, Fehmi Tabak

**Affiliations:** 1 Department of Infectious Diseases and Clinical Microbiology, Antalya Training and Research Hospital, Antalya, Turkey; 2 Department of Infectious Diseases, İstanbul Education Research Hospital, İstanbul, Turkey; 3 Department of Infectious Diseases and Clinical Microbiology, Çukurova University Faculty of Medicine, Adana, Turkey; 4 Department of Infectious Diseases and Clinical Microbiology, Mustafa Kemal University Faculty of Medicine, Hatay, Turkey; 5 Department of Gastroenterology, Medeniyet University Göztepe Training and Research Hospital, İstanbul, Turkey; 6 Department of Infectious Diseases, İstanbul Şişli Hamidiye Etfal Training and Research Hospital, İstanbul, Turkey; 7 Department of Infectious Diseases, Haseki Education Research Hospital, İstanbul, Turkey; 8 Department of Infectious Diseases and Clinical Microbiology, Harran University Faculty of Medicine, Şanlıurfa, Turkey; 9 Department of Infectious Diseases, İstanbul Doctor Lütfi Kırdar Kartal Training and Research Hospital, İstanbul, Turkey; 10 Department of Infectious Diseases and Clinical Microbiology, Namık Kemal University Faculty of Medicine, Tekirdağ, Turkey; 11 Department of Infectious Diseases and Clinical Microbiology, Ankara Yıldırım Beyazıt University Faculty of Medicine, Ankara, Turkey; 12 Department of Infectious Diseases and Clinical Microbiology, Ege University Faculty of Medicine, İzmir, Turkey; 13 Department of Infectious Diseases, İzmir Tepecik Training and Research Hospital, İzmir, Turkey; 14 Department of Infectious Diseases and Clinical Microbiology, Eskişehir Osmangazi University Faculty of Medicine, Eskişehir, Turkey; 15 Department of Infectious Diseases and Clinical Microbiology, Düzce University Medical School, Düzce, Turkey; 16 Department of Infectious Diseases and Clinical Microbiology, Karadeniz Teknik University Faculty of Medicine, Trabzon, Turkey; 17 Department of Infectious Diseases and Clinical Microbiology, İstanbul University-Cerrahpaşa, Cerrahpaşa Faculty of Medicine, İstanbul, Turkey; 18 Department of Infectious Diseases and Clinical Microbiology, Ondokuz Mayıs University Faculty of Medicine, Samsun, Turkey; 19 Department of Infectious Diseases, Samsun Education Research Hospital, Samsun, Turkey; 20 Department of Infectious Diseases and Clinical Microbiology, Recep Tayyip Erdoğan University Training and Research Hospital, Rize, Turkey; 21 Department of Infectious Diseases and Clinical Microbiology, Akdeniz University Medical School, Antalya, Turkey; 22 Department of Infectious Diseases and Clinical Microbiology, Gaziosmanpaşa University Medical Faculty, Tokat, Turkey; 23 Department of Infectious Diseases and Clinical Microbiology, Gaziantep University Medical Faculty, Gaziantep, Turkey; 24 Department of Infectious Diseases, İstanbul Bağcılar Training and Research Hospital, İstanbul, Turkey; 25 Department of Infectious Diseases and Clinical Microbiology, Çanakkale 18 Mart University Faculty of Medicine, Çanakkale, Turkey; 26 Department of Infectious Diseases and Clinical Microbiology, Gazi University Faculty of Medicine, Ankara, Turkey; 27 Department of Infectious Diseases and Clinical Microbiology, Hitit University Faculty of Medicine, Çorum, Turkey; 28 Department of Infectious Diseases, Ankara Dr. Abdurrahman Yurtaslan Ankara Oncology Training and Research Hospital, Ankara, Turkey; 29 Department of Infectious Diseases, Ankara Numune Training and Research Hospital, Ankara, Turkey; 30 Department of Gastroenterology, Bolu İzzet Baysal University Medical Faculty, Bolu, Turkey; 31 Department of Infectious Diseases, Health Science University Gülhane Faculty of Medicine, Ankara, Turkey; 32 Department of Infectious Diseases and Clinical Microbiology, Fırat University School of Medicine, Elazığ, Turkey; 33 Department of Infectious Diseases and Clinical Microbiology, Zonguldak Bülent Ecevit University Training and Research Hospital, Zonguldak, Turkey; 34 Department of Infectious Diseases and Clinical Microbiology, Yüzüncü Yıl University Faculty of Medicine, Van, Turkey; 35 Department of Infectious Diseases and Clinical Microbiology, Sakarya University Training and Research Hospital, Sakarya, Turkey; 36 Department of Infectious Diseases, İstanbul Bakırköy Dr. Sadi Konuk Training and Research Hospital, İstanbul, Turkey; 37 Department of Infectious Diseases and Clinical Microbiology, Bolu İzzet Baysal University Medical Faculty, Bolu, Turkey

**Keywords:** Drug therapy, drug users, hepatitis C virus

## Abstract

**Background:**

People who inject drugs (PWID) should be treated in order to eliminate hepatitis C virus in the world. The aim of this study was to compare direct-acting antivirals treatment of hepatitis C virus for PWID and non-PWID in a real-life setting.

**Methods:**

: We performed a prospective, non-randomized, observational multicenter cohort study in 37 centers. All patients treated with direct-acting antivirals between April 1, 2017, and February 28, 2019, were included. In total, 2713 patients were included in the study among which 250 were PWID and 2463 were non-PWID. Besides patient characteristics, treatment response, follow-up, and side effects of treatment were also analyzed.

**Results:**

Genotype 1a and 3 were more prevalent in PWID-infected patients (20.4% vs 9.9% and 46.8% vs 5.3%). The number of naïve patients was higher in PWID (90.7% vs 60.0%), while the number of patients with cirrhosis was higher in non-PWID (14.1% vs 3.7%). The loss of follow-up was higher in PWID (29.6% vs 13.6%). There was no difference in the sustained virologic response at 12 weeks after treatment (98.3% vs 98.4%), but the end of treatment response was lower in PWID (96.2% vs 99.0%). In addition, the rate of treatment completion was lower in PWID (74% vs 94.4%).

**Conclusion:**

Direct-acting antivirals were safe and effective in PWID. Primary measures should be taken to prevent the loss of follow-up and poor adherence in PWID patients in order to achieve World Health Organization’s objective of eliminating viral hepatitis.

**Main Points**

- Our study is one of the few studies presenting differences in demographic characteristics and treatment responses between people who inject drugs (PWID) and non-PWID in our country.
- We found a nonsignificant difference in the sustained virologic response at 12 weeks after the treatment rate of PWID and non-PWID.
- The end of treatment response was lower in PWID.
- In addition, the rate of treatment completion was lower in PWID.
- Direct-acting antivirals were safe and effective in PWID. Primary measures should be taken to prevent the loss of follow-up and poor adherence in PWID patients.

## Introduction

Hepatitis C virus (HCV) is the etiologic agent of chronic hepatitis C (CHC) and a major cause of cirrhosis, liver cell failure, and hepatocellular carcinoma.^1^ According to the latest data of World Health Organization (WHO), the number of people infected with HCV is approximately 71 million people.^2^ People who inject drugs (PWID) are one of the risk groups who are at higher risk of HCV infection. It is estimated that around 10 million (7.5 million with chronic HCV infection) PWID are infected with HCV worldwide.^3^ Furthermore, WHO estimated that 52% of the 15.6 million global PWID have evidence of hepatitis C exposure.^2^ In addition to the complications of liver disease, healthcare costs continue to rapidly grow with increasing HCV infections in PWID.^4,5^

Hepatitis C virus seroprevalence is about 0.6-1% in Turkey, whose total population is around 80 million.^6,7^ On the other hand, according to recently published studies, anti-HCV positivity in PWID has reached 50.1% in inpatients treated in the alcohol and drug addiction treatment centers.^8^

Chronic hepatitis C has recently become a curable disease as a result of new direct-acting antivirals (DAAs).^9^ But it may be difficult to access DAAs in low- and middle-income countries.^10,11^ The ease of use for a new treatment is a driving force for the WHO initiate that aims to develop a strategy to eliminate HCV as a major public health threat by 2030. Specific targets include an 80% reduction in new infections, a 65% reduction in HCV-related deaths, and 80% of the HCV-infected population in treatment.^12^ In Turkey, DAAs have been available for the treatment of patients with CHC since June 2016 by the Turkish Drug Administration.^13,14^

People who inject drugs (PWID) are a risk population who are harder to access; additionally, non-adherence to treatment and post-treatment reinfection rates are higher in PWID compared to other risk populations.^15^ During the interferon period, the treatment of these patients was difficult due to many side effects and long treatment periods.^16^ Current guidelines indicate that PWID should be treated for reducing transmission of HCV.^17,18^ However, populations at greatest risk for new infections have the highest risk of not receiving treatment due to stigmatization.^19^

The characteristics of HCV infection in intravenous drug use are different from that in the general population.^20^ Hepatitis C virus genotypes (GTs) may differ in this population in addition to factors reducing access to treatment by intravenous drug users such as social factors and comorbid psychiatric disorders.^21,22^ Genotype 3 was seen more frequently in PWID than the general population (58.6% vs 11.5%).^23,24^

The aim of this multicenter and prospective study was to compare clinical and demographic characteristics of chronic HCV infection and the efficacy of HCV infection by DAAs among PWID and non-PWID in a real-life setting in Turkey.

## MATERIALS AND METHODS

### Study Design

This study was a prospective, non-randomized, observational multicenter cohort study, in 37 centers distributed geographically across Turkey. All patients treated with DAAs therapy between April 1, 2017, and February 28, 2019, were included. In total, 2713 patients were included in the study, among which 250 were PWID (9.2%) and 2463 were non-PWID (90.8%).

Infectious Diseases and Clinical Microbiology Specialty Society (EKMUD) and the Turkish Viral Hepatitis Society (VHSD) created an online database and collected data on CHC patients on DAAs treatment in Turkey. Data were collected on-site by physicians who were responsible for the treatment of patients. Centers included in this study covered all regions of Turkey such as Marmara, Mediterranean, Central Anatolia, Aegean, Black Sea, Eastern Anatolia, and Southeastern Anatolia.

### Study Population

Patients over 18 years of age with HCV RNA positive test for at least 6 months prior to screening under DAAs were enrolled in this observational study. People who inject drugs were defined as a person who had used intravenous drugs at least once in their life. The Turkish reimbursement criteria were applicable for therapy indication and DAAs regimen choice on all these patients.^13,14^ Health implementation guideline of Turkey in the treatment of HCV infection with DAA is shown in Table 1. Liver biopsies were scored for grading and staging according to Knodell’s modified system.^25^ End of treatment response (ETR) was defined as the number of patients whose HCV RNA could not be detected at the end of the treatment. Sustained viral response (SVR) was also defined as the number of patients whose HCV RNA could not be detected at 12 weeks after the end of therapy.

### Endpoints of the Study

We studied the treatment uptake of PWID and non-PWID in Turkey. The primary endpoints were viral clearance at the completion of DAAs treatment and at 12 weeks after treatment (SVR12). Besides, patient characteristics and side effects of treatment were also analyzed.

### Statistical Analysis

All analyses were performed using Statistical Package for the Social Sciences version 21 (IBM Corp.; Armonk, NY, USA). Descriptive statistics of patient characteristics were presented for continuous variables, means and SD with range, for categorical variables, proportions, and percentages. Independent *t*-tests were used for the comparison of 2 continuous variables. Chi-square tests were used for comparing categorical variables. A *P* value less than .05 was considered statistically significant.

## Results

### Baseline Characteristics

The characteristics of 2713 patients were described in Table 2. People who inject drugs were significantly younger and predominantly male. They had a similar body mass index, and GT 1a and GT 3 were observed more in PWID infected with HCV, whereas GT 1b was the most prevalent in non-PWID. Alanine aminotransferase levels were higher in PWID compared to non-PWID. There was no significant difference in viral load. The number of naive patients was higher in PWID. While the median fibrosis stages were similar in each group, fibrosis 4 and 5 stages were higher in non-PWID, and fibrosis 2 stage was higher in PWID. The number of patients with cirrhosis was higher in non-PWID than PWID. There was no significant difference between the groups considering HBV and HIV coinfections in the study population.

## Antiviral Treatment

**Figure 1.**
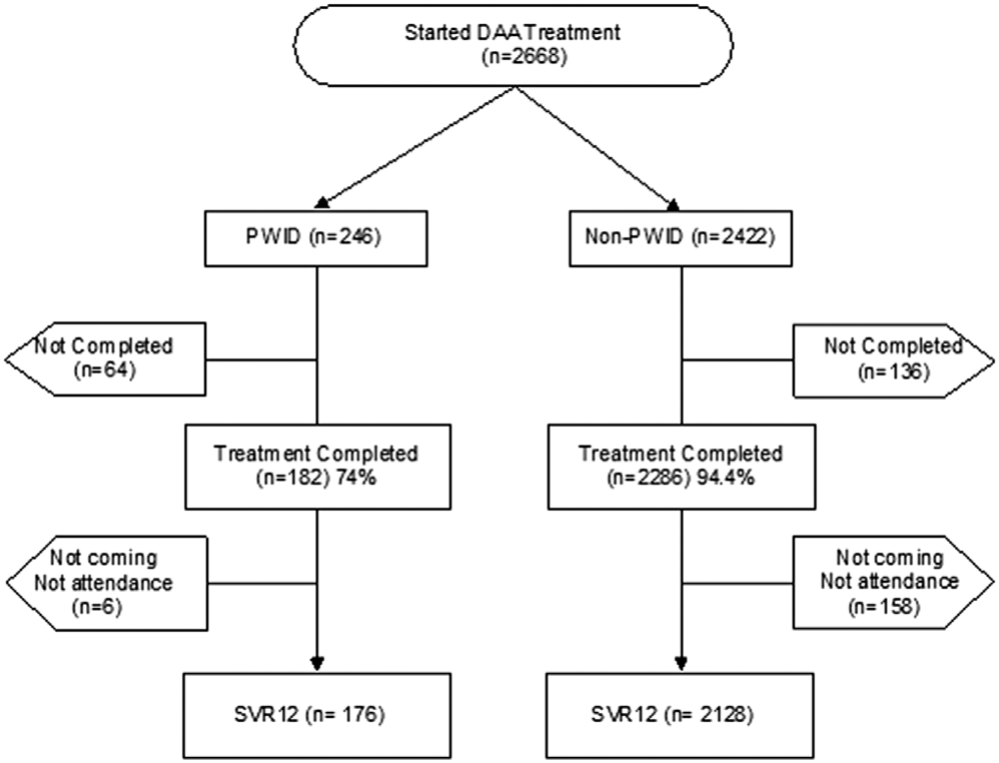
Treatment flow chart.

Due to the lack of treatment data in 45 patients, the antiviral treatment was evaluated on 2668 (246 PWID, 2422 non-PWID) patients in Table 3. Besides the regimens approved to use in Turkey by the Turkish reimbursement agency, 3 different regimes were used among non-PWID patients. These regimes included sofosbuvir 400 mg + daclatasvir 60 mg or sofosbuvir 400 mg + velpatasvir 100 mg which were obtained from abroad and self-supplied by the patients. Since glecaprevir 100 mg + pibrentasvir 40 mg has been approved and included in the reimbursement list by the Turkish government in January 2019, 28 patients in the study used this regimen. Compared to sofosbuvir + ribavirin used predominantly in GT 3 patients, ombitasvir + paritaprevir/r + dasabuvir (3D) + ribavirin use was more prevalent in GT 1a and hence observed more in the treatment of PWID patients in this study. The loss of follow-up was higher in PWID. There was no difference in the SVR12; however, ETR was lower in PWID. There was no significant difference in the rate of side effects between PWID and non-PWID; only fatigue was significantly higher in non-PWIDs. The treatment chart is shown in Figure 1.

**Figure 2.**
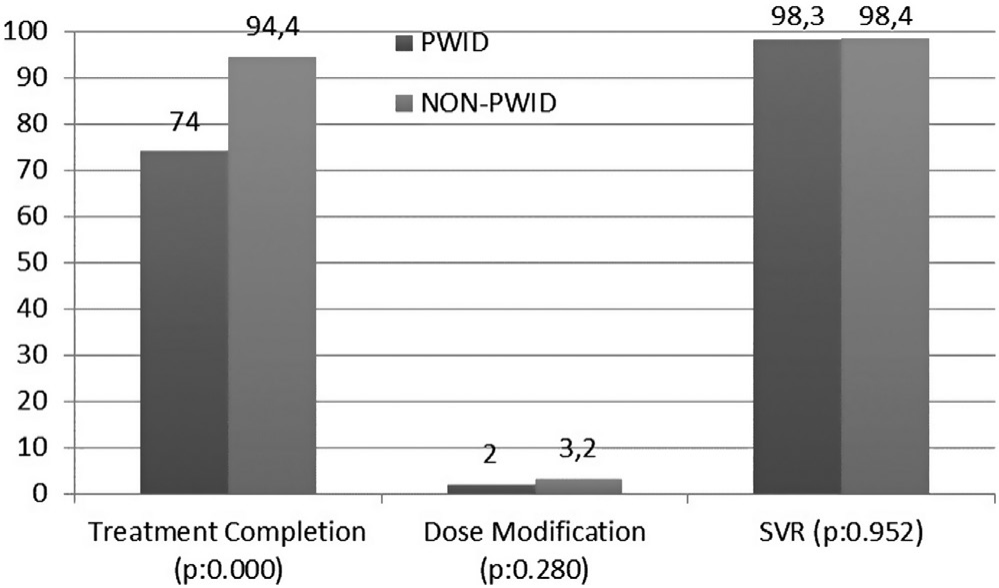
Outcome of antiviral therapy between PWID and non-PWID. SVR, sustained virologic response 12 weeks after treatment completion; PWID, people who inject drugs.

The outcome of antiviral treatment is presented in Figure 2. There was no significant difference in treatment modification and SVR. The rate of treatment completion was lower in PWID.

## Discussion

According to this multicenter cohort study conducted in Turkey, PWID with CHC was 9.2% among all CHC patients. These data could be employed in developing strategies to prevent and eradicate HCV infection in Turkey as PWID are one of the high-risk populations for HCV. For people who inject drugs in CHC patients, seroprevalence was reported between 19.8% and 50% in other countries.^26,27^ In Eastern Europe, including neighboring countries of Turkey such as Georgia, Ukraine, and Russia, PWID constitutes relatively higher proportions of HCV infections as 25.6%, 21.5%, and 40.4% of the total infections, respectively.^28^ Our study presented the magnitude of PWID status in CHC in Turkey and revealed that PWID prevalence in CHC could be considered as lower than the other countries. The results of our study showed that we could achieve WHO targets by 2030 with less effort potentially compared to other countries if we take the necessary measures.

Our results showed that PWID were younger (30 ± 10 years vs. 57 ± 14 years, *P* < .00001), and the majority of them were younger than 30 years old (65.6%). The rate of males was higher (94.0% vs 46.5%, *P* < .00001) in PWID. The fibrosis stage and the frequency of cirrhosis were lower in PWID due to the younger age of our study group. Among all the patients, the most prevalent genotype was GT 1; GT 1a and GT 3 were higher in PWID. Our data were consistent with other PWID HCV studies.^26,29-31^ Although observing new HCV infections in the younger populations more frequently is alarming, there is one benefit of changing demographic in that these individuals were typically younger, less likely to have cirrhosis, and more likely treatment naive. This benefit would suggest that they were possibly easier to treat. However, it is still difficult to access this population because they were actively using drugs, having irregular lives, and social disorders.^26,32^

Due to poor adherence, adverse events, and high re-infection rates, PWID was notoriously harder to treat before DAAs.^23,34^ The first recommendations for treatment of PWID with CHC were published in 2013.^35^ International guidelines have been updated for CHC therapy among PWID in 2015,^36^ after these updates, there were many studies published about the treatment success of PWID in CHC.^29-31^ Our study is one of the few studies presenting differences in demographic characteristics and treatment responses between PWID and non-PWID. We found a nonsignificant difference in the SVR12 rate of PWID and non-PWID (98.4% vs 98.3%, *P* = .952). Despite the presence of a large number of GT3 patients, SVR in our study was quite high because sofosbuvir + ribavirin and sofosbuvir + ledipasvir regimens are less effective on GT3 HCV than other genotypes.^37,38^

In our study, ETR was lower (96.2% vs 99.0%, *P* = .001) and the loss of follow-up was higher (29.6% vs 13.6%, *P* = .000) in PWID than non-PWID. Adherence assessments should consider missed doses and treatment discontinuation due to poor treatment adherence in PWID.^35,39^ Compared to the other studies, we found that the rate of completion of the treatment in this study was lower.^40,41^ In most of these studies, opiate substitution therapy (OST) was implemented for PWID as it improves adherence to treatment and reduces the rate of re-infection in PWID.^29,42^ Unfortunately, in our study, these data were missing. Although the ETR was low, the SVR12 of PWID in our study was found to be unaffected and quite high. These data suggest that the period between ETR and SVR12 is important for maintaining engagement in post-treatment care and follow-up. At the beginning of the study, treatment of 246 PWID was initiated. However, 70 patients were lost to follow-up, and only 176 patients have reached SVR12. Patients who did not complete the treatment may continue to transmit HCV. This multicenter study showed that, in Turkey, PWID should be under direct observation during HCV infection treatment. However, due to nonadherence to the therapy, the potential risk of transmission should be considered by the public health practitioners and policy makers, as these patients are the key to viral elimination. Therefore, strategies to keep PWID patients in continuous retention should be developed to decrease HCV infections among PWID and to increase treatment adherence, retention, and follow-up in HCV treatment.

In this study, the coinfection rates of HCV and HBV were 6.9% in PWID and 4.1% in non-PWID. In the Belgian cohort study, these rates were 4.2% and 0.8%, respectively.^26^ On the other hand, they found that HCV and HIV coinfections rates were 12.5% and 6.9% in PWID and non-PWID as opposed to our study where the coinfection rates of HCV and HIV were 2.5% and 1% in PWID and non-PWID. These rates were highly correlated to the epidemiology of infections and different characteristics of the HCV epidemic in each country, and as a result, it could be different in many studies.^43,44^ Overall, we had lower HCV and HIV co-infection rates in PWID compared to other studies. Both HIV-HBV and HIV-HCV coinfections increase the morbidity and mortality caused by each disease, as well as significantly complicate the burden on medical management and health systems.^45-47^

Our study has several limitations. These limitations include that percentage of people with active drug use and on OST was not known. Because this study is a large observational prospective, multi-center cohort study, we could not reach some data in the subgroup analysis.

## Conclusion

Primary measures should be taken to prevent the loss of follow-up and poor adherence in patients in the countries such as Turkey, in order to prevent increasing HCV infections in PWID. Our results confirmed that strategies to support patient’s retention until SVR are required. New policies for preventing new infections among PWID should be developed by the public health organizations in the government and in the non-governmental bodies in Turkey to achieve WHO’s objectives of preventing and eliminating viral hepatitis.

## Figures and Tables

**Table 1.** Health Implementation Guideline of Turkey in the Treatment of HCV Infection with Direct-Acting Antivirals

**HCV Genotype**	**Non-cirrhosis (F1-3)**		**Compensated Cirrhosis (F4-6) (Child Pugh A)**		**Decompensated Cirrhosis (F4-6) (Child Pugh B, C)**	
	**Native**	**Experienced**	**Naive**	**Experienced**	**Naive**	**Experienced**
**1a**	3D+RBV-12 wk	LDV/SOF+RBV-12 wk LDV/SOF-24 wk 3D+RBV-12wk*	3D+RBV-24 wk	LDV/SOF+RBV-12 wk LDV/SOF-24 wk 3D+RBV-12 wk*	LDV/SOF+RBV-12 wk LDV/SOF-24 wk	LDV/SOF+RBV-12 wk LDV/SOF-24 wk
**1b**	3D-12wk	LDV/SOF+RBV-12 wk LDV/SOF-24 wk 3D-12 wk*	3D-12 wk	LDV/SOF+RBV-12 wk LDV/SOF-24 wk 3D-12 wk*	LDV/SOF+RBV-12 wk LDV/SOF-24 wk	LDV/SOF+RBV-12 wk LDV/SOF-24 wk
**2**	SOF+RBV-12 wk	SOF+RBV-12 wk	SOF+RBV-12 wk	SOF+RBV-12 wk	SOF+RBV-12 wk	SOF+RBV-12 wk
**3**	SOF+RBV-24 wk	SOF+RBV-24 wk	SOF+RBV-24 wk LDV/SOF-24 wk	SOF+RBV-24 wk LDV/SOF-24 wk	SOF+RBV-24 wk	SOF+RBV-24 wk LDV/SOF-24 wk
**4**	OBV+PTV+RBV-12 wk	LDV/SOF+RBV-12 wk LDV/SOF-24 wk OBV+PTV+RBV-12 wk*	OBV+PTV+RBV-12 wk	LDV/SOF+RBV-12 wk LDV/SOF-24 wk OBV+PTV+RBV-12 wk*	LDV/SOF+RBV-12 wk LDV/SOF-24 wk	LDV/SOF+RBV-12 wk LDV/SOF-24 wk

*Except for previously treated with protease inhibitors, and patients with Child-Pugh B or C cirrhosis.

LDV, ledipasvir; OBV, ombitasvir; RBV, ribavirin; PTV, paritaprevir; wk, week; 3D, ombitasvir + paritaprevir/ritonavir + dasabuvir; HCV, hepatitis C virus.

**Table 2.** Demographic and Baseline Characteristics of the Patients

	PWID (n = 250)	Non-PWID (n = 2463 )	*P*
Male, % (n)	94.0% (235)	46.5% (1145)	.000
Age (years, mean ± SD) (range)	30 ± 10 (18-77)	57 ± 14 (18-97)	.000
<30	164 (65.6%)	160 (6.5%)	
31-60	80 (32%)	1199 (44.8)	
>61	6 (2.4%)	1104 (44.8%)	
BMI (mean ± SD) (range)	24.63 ± 2.77 (19.03-32.15)	26.34 ± 4.54 (15.23-49.95)	.902
HCV GT, % (n)			.000
GT1	88 (35.2)	2177 (88.4)	
GT1a	51 (20.4)	244 (9.9)	
GT1b	31 (12.4)	1835 (74.5)	
GT1-undermined	6 (2.4)	97 (3.9)	
GT2	21 (8.4)	84 (3.4)	
GT3	117 (46.8)	130 (5.3)	
GT4	22 (8.8)	62 (2.5)	
GT5	1 (0.4)	8 (0.3)	
GT-undermined	1 (0.4)	2 (0.1)	
ALT level, baseline median, IU/mL (range)	66 (range: 10.5-588)	39 (range: 6.5-841)	.000
HCV RNA load, baseline median, IU/mL (range)	639 000 (range: 84-73 200 000	963 000 (range 24-962 224 453)	.077
Treatment status, n (%)			
Naive patient	223 (90. 7)	1447 (60.0)	.000
PegIFN experienced	22 (95.7)	875 (91.5)	
Boc + PegIFN + RBV	1 (4.3)	35 (3.7)	
Tel + PegIFN + RBV	-	46 (4.8)	
Biopsy status, median score (range), n (%)			
Carried liver biopsy	136 (55.3)	1343 (56.8)	.646
Fibrosis	2(0-6),134 (98.5)	2 (0-6), 1330 (99)	.004
HAI score	7 (1-14),136	7 (1-18),1303(97)	.458
Patient with cirrhosis, n (%)	9 (3.7)	325 (14.1)	.000
Child Pugh A	8 (88.9)	276 (87.6)	
Child Pugh B-C	1 (11.1)	39 (12.4)	
Coinfections, n (%)			
HBsAg	15/216 (6.9)	97/2354 (4.1)	.56
HIV	5/200 (2.5)	20/1984 (1.0)	.72

BMI, body mass index (kg/m); ALT, alanine aminotransferase; GT, genotype; Boc, boceprevir; HBsAg, hepatitis B surface antigen; HCV RNA, hepatitis C virus RNA; HIV, human immunodeficiency virus; PegIFN, pegylated interferon; RBV, ribavirin; Tel, telaprevir; HCV, hepatitis C virus; SD, standard deviation; PWID, people who inject drugs; HAI, histological activity index.

**Table 3.** Antiviral Treatment Characteristics, Treatment Responses to DAA in PWID Versus Non-PWID

	PWID (n = 246)	Non-PWID (n = 2422)	*P*
Type of treatment: n, (%)			.000
Ombitasvir + paritaprevir/r + dasabuvir	22, (8.9)	1129 (46.6)	
Ledipasvir + sofosbuvir	34 (13.8)	626 (25.8)	
Sofosbuvir + ribavirin	120 (48.8)	167 (6.9)	
Paritaprevir + ritonavir + ombitasvir + dasabuvir + ribavirin	36 (14.6)	226 (9.3)	
Ledipasvir + sofosbuvir + ribavirin	9 (3.7)	208 (8.6)	
Paritaprevir + ritonavir + ombitasvir + ribavirin	16 (6.5)	33 (1.4)	
Paritaprevir + ritonavir + ombitasvir	-	10 (0.4)	
Sofosbuvir + daklatasvir	-	2 (0.1)	
Sofosbuvir + velpatasvir	-	1 (0.0)	
Sofosbuvir	-	1 (0.0)	
Glecaprevir + pibrentasvir	9 (3.7)		
Lost of follow-up, n (%)	74 (29.6)	335 (13.6)	.000
Treatment responses, n (%)			
ETR	175/182 (96.2)	2263/2286 (99.0)	.001
SVR12	173/176 (98.3)	2093/2128 (98.4)	.952
Side effects: (any), n (%)			
Fatigue	5 (2.0)	188 (7.6)	.001
Pruritus	5 (2.0)	144 (5.8)	.011
Headache	2 (0.8)	60 (2.4)	.099
Insomnia	6 (2.4)	67 (2.7)	.766
Nausea	3 (1.2)	74 (3.0)	.102
Arthralgia/myalgia	-	23 (0.9)	.125

ETR, end of treatment response; SVR, sustained viral response; PWID, people who inject drugs; DAA, direct-acting antiviral.
